# An Account of Diseases from the 20th of October to the 20th of November, 1799

**Published:** 1799-12

**Authors:** 


*An Account of Difeafes from the 20th of October to the 20th of November,* 1799.

| | No. of Cafes. | | No. of Cafes. |
|---|---|---|---|
| ACUTE DISEASES. | | Peripneumony - - | 3 |
| Contagious malignant Fever | 22 | Peritoneal Inflammation - | 2 |
| Scarlet Fever - - | 15 | Acute Rheumatifm - | 7 |
| Meafles - - - - - | 12 | Ophthalmia - - - | 2 |
| Catarrh - - - | 18 | Angina - - - | 2 |
| | | Eryfipelas | |

| | No. of Cases. | | No. of Cases |
|---|---|---|---|
| Eryfipelas - - - | 1 | Enterodynia - - | 5 |
| Acute Difeafes of Infants | 8 | Hæmorrhoids - - | 3 |
| Hooping Cough - - | 3 | Worms - - - - | 2 |
| Childbed and Milk Fevers | 5 | Chlorofis and Amenorrhœa | 11 |
| Hectic and Slow Fever - | 7 | Menorrhagia - - | 2 |
| CHRONIC DISEASES. | | Fluor Albus - - | 3 |
| Cough and Difpnœa - | 32 | Prolapfus Uteri - - | 1 |
| Hæmoptoe - - - | 3 | Scirrhus Uteri - - | 2 |
| Pulmonary Confumption - | 6 | Gravel Dyfury - - - | 3 |
| Pleurodyne - - - | 3 | Scirrhus of the Liver - | 1 |
| Chronic Rheumatifm - | 14 | Jaundice - - - | 2 |
| Afthenia - - - | 16 | Tabes Mefenterica - - | 2 |
| Dropfy - - - | 6 | Rickets - - - - | 4 |
| Cephalea - - - | 4 | Scrophula - - - | 6 |
| Vertigo - - - | 2 | Lepra - - - | 2 |
| Epilepfy - - - | 3 | Itch and Prurigo - - | 10 |
| Hyfteria - - - | 1 | Impetigo - - - | 3 |
| Palfy - - - - | 2 | Nettle Rafh - - - | 1 |
| Hydrocephalus - - | 1 | Herpes - - - | 1 |
| Palpitatio - - - | 1 | Acne - - - - | 5 |
| Dyfpepfia - - - | 11 | Dandriff - - - | 2 |
| Gaftrodynia - - - | 7 | Porrigo - - - - | 3 |
| Hæmatemefis - - | 2 | Erythema - - - | 1 |
| Bilious Vomiting, and Diarrhœa - - - | 13 | Purpura - - - - | 1 |
| | | Lupus - - - - | 1 |

The meafles, though extenfively diffufed, have continued mild and moderate. The fcarlet fever has increafed, fince the laft report, both in extent and in the violence of its fymptoms: but the typhus, or contagious malignant fever, has been the moft frequent, as well as the moft fatal, of all acute difeafes. Of the number fpecified in the lifts for the prefent and preceding month, ten patients died, whereas the ufual proportion of deaths from this feve , weftward of Temple Bar, is one in feventeen or eighteen cafes. The habitations of the poor, within or adjoining to the city, have fuffered moft; and fome, I am informed, have been nearly depopulated, the infection having extended to every inmate. The rumour of a plague was totally devoid of foundation: one of the perfons faid to have been affected with it from opening fome bales of prize-cotton, died with the ufual fymptoms of a peripneumony. It was afterwards afcertained, by anatomical diffection, that his death was occafioned by a violent inflammation of the lungs, which feemed to have been brought on by intemperance in drinking, and expofure to a cold and damp air, at an unfeafonable time of the night.

The typhus, or contagious malignant fever, was, in September, attended with a dull pain of the head, great debility, or a fenfe of laffitude, tude, and pains referred to the bones, tremblings, reftleffnefs with flight delirium, a querulous tone of voice, a fmall and frequent pulfe, heat of the fkin, thirft, a fur upon the tongue, firft of a dirty white colour, but turning, in the latter ftage of the difeafe, to a yellowifh brown. In this form the fever continued thirteen days without any dangerous fymptom, and then fuddenly difappeared, leaving the patient for fome time after languid and difpirited. All the individuals of a family were fucceffively affected with the fame train of fymptoms, many of them fo flightly as not to be much confined to their beds. To this contagious fever alone, Dr. CULLEN ought to have applied the denomination of typhus mitior: he has improperly comprifed under it the flow or nervous fever defcribed by HUXHAM and GILCHRIST, which may rather be confidered as a fpecies of hectic, and is not received by infection.

In October and November, the difeafe, as is ufual, affumed its more dangerous form. The pain of the head was deep feated, and attended with great confufion of ideas; a total lofs of ftrength fuddenly took place, and the limbs felt fore, as if they had been all over bruifed. The pulfe was weak and irregular: a thick, fordid, brown fur covered all the upper part of the tongue; the tongue itfelf became hard, dry, and almoft immoveable; and the teeth were alfo covered with a brown or black cruft. There was a fmarting or burning heat of the fkin, which conveyed an unpleafant, benumbing fenfation to the fingers and wrift of the practitioner who felt the pulfe. The eyes were frequently fuffufed; the head-ach terminated, during the fecond week, in coma or ftupor, with great infenfibility, deafnefs, &c. Thefe fymptoms were, however, more favourable than a ftate of agitation and watchfulnefs. In the fatal cafes there occurred, a few hours before death, a laborious refpiration, with a fluttering, irregular pulfe, difficulty of fwallowing, and fometimes hiccough. A favourable crifis was made by fweating, accompanied, in fome inftances, by a fenfation of coldnefs: a diarrhœa took place only in one patient. The critical days feemed to be, the feventh, the eleventh, the thirteenth, the fifteenth, and the nineteenth, but the moft numerous crifes were on the eleventh and thirteenth. The changes, whether for recovery or death, took place very fuddenly. I did not obferve petechial fpots in any of the cafes; nor the alternations of cold fhiverings with flufhes of heat, which moft practical writers defcribe as the primary fymptoms of malignant fever.

The Peruvian bark was of no confiderable advantage in the fever above above described, unless emetics had been administered before the end of the fourth day. Several of the patients were washed twice a day with cold water and vinegar, with only temporary relief. In the most unfavourable state of the disease, blisters were of great utility; they produced a remission of the fever, made the pulse more free and regular, and seemed to be the means of procuring rest. Some patients were relieved by them in whom the loss of sight, of speech, and of the power of deglutition seemed to threaten immediate dissolution. If two or three persons lie in one bed, which frequently happens in the crowded dwellings of the poor, some one almost certainly falls a victim to the fever; I am, however, happy to state, that a man and his wife, who had the fever in its most malignant form, and were confined to the same bed, have been restored during the present month, by the active exertions of a medical friend and assistant.*

The state of the atmosphere must have undoubtedly caused the great extension and aggravated symptoms of the malignant fever. On this subject it is proper to remark, that between the 22d of June and the 17th of November, in a period of 147 days, there were only eight days free from rain; a circumstance, perhaps, unparalleled in meteorological observations.

R. W.

*Account of Diseases in an Eastern District of London, from the 20th of October to the 20th of November.*

